# Development and validation of a nomogram for predicting histologic subtypes of subpleural non-small cell lung cancer using ultrasound parameters and clinical data

**DOI:** 10.3389/fonc.2024.1477450

**Published:** 2024-11-08

**Authors:** Feng Mao, Mengjun Shen, Yi Zhang, Hongwei Chen, Yang Cong, Huiming Zhu, Chunhong Tang, Shengmin Zhang, Yin Wang

**Affiliations:** ^1^ Department of Medical Ultrasound, The First Affiliated Hospital of Ningbo University, Ningbo, China; ^2^ Department of Ultrasound, Shanghai Pulmonary Hospital, School of Medicine, Tongji University, Shanghai, China

**Keywords:** non-small cell lung cancer, subpleural pulmonary lesion, nomogram, ultrasound, contrast-enhanced ultrasound

## Abstract

**Aims:**

To develop and validate an individualized nomogram for differentiating the histologic subtypes (adenocarcinoma and squamous cell carcinoma) of subpleural non-small cell lung cancer (NSCLC) based on ultrasound parameters and clinical data.

**Methods:**

This study was conducted retrospectively between March 2018 and December 2019. Patients were randomly assigned to a development cohort (DC, n=179) and a validation cohort (VC, n=77). A total of 7 clinical parameters and 16 ultrasound parameters were collected. Least absolute shrinkage and selection operator regression analysis was employed to identify the most significant predictors utilizing a 10-fold cross-validation. The multivariate logistic regression model was applied to investigate the relevant factors. An individualized nomogram was then developed. Receiver operating characteristic (ROC) curve, calibration plot and decision curve analysis (DCA) were applied for model validation in both DC and VC.

**Results:**

Following the final regression analysis, gender, serum carcinoembryonic antigen, lesion size and perfusion defect in contrast-enhanced ultrasound were entered into the nomogram. The model showed moderate predictive ability, with an area under the ROC curve of 0.867 for DC and 0.838 for VC. The calibration curves of the model showed good agreement between actual and predicted probabilities. The ROC and DCA curves demonstrated that the nomogram exhibited a good predictive performance.

**Conclusion:**

We developed a nomogram that can predict the histologic subtypes of subpleural NSCLC. Both internal and external validation revealed optimal discrimination and calibration, indicating that the nomogram may have clinical utility. This model has the potential to assist clinicians in making treatment recommendations.

## 1 Introduction

Lung cancer is one of the most common malignant tumors and the leading cause of cancer-related death worldwide, with 2,206,771 newly diagnosed cancer cases and 1,796,144 cancer-related deaths in 2020 (1). Approximately 85% of lung cancers are classified as non-small cell lung cancer (NSCLC), of which lung adenocarcinoma (LUAD) and lung squamous cell carcinoma (LUSC) are the two major histologic subtypes (2–4). Recent advances in targeted therapies highlight the importance of the accurately distinguishing between the two subtypes of NSCLC (5, 6). In clinical practice, the pathological examination based on biopsy or surgical resection is the gold standard for the differential diagnosis of LUAD and LUSC. However, this method is mainly limited by the inherent risk of invasive procedures and sampling errors (7–9). An effective non-invasive alternative is therefore needed to aid in the prognostication of the two subtypes of NSCLC.

Although chest computed tomography (CT) is the main method for diagnosing pulmonary lesions, many of them remain indeterminate after CT imaging analysis (10). Approximately 40% of lung cancers present as peripheral lung masses, usually involving the pleura, and are therefore potentially visible on ultrasound (US) (11). In recent years, with the development of US techniques, especially contrast-enhanced ultrasound (CEUS), the diagnostic accuracy of subpleural pulmonary lesions on ultrasound has been advanced rapidly (12, 13). Because of its convenience, radiation-free and real-time monitoring, US has become a crucial complementary technique for the imaging diagnosis of subpleural pulmonary lesions.

Uptoday, several studies have delineated the ability diagnose subtypes of NSCLC by radiographic imaging patterns alone, or in combination with clinical data (14–16). The preponderance of research in this domain is centered on CT and positron emission tomography (PET) scans. However, the diagnostic ability of ultrasound (US) parameters combined with clinical data to distinguish subpleural NSCLC histological subtypes remains unconfirmed. Therefore, the objective of this study was to develop a predictive model for the histologic subtypes (LUSC and LUAD) of subpleural NSCLC based on US parameters and clinical data.

## 2 Materials and methods

This retrospective cohort study was in accordance with the ethical standards formulated in the Helsinki Declaration and approved by the Institutional Review Board (No. K18-197Y). Moreover, our study protocol followed the statement of Transparent Reporting of a Multivariable Prediction Model for Individual Prognosis or Diagnosis (TRIPOD) and was registered in the Chinese Clinical Trial Registry (No. ChiCTR1800019828). All patients signed a written informed consent to participate in this research.

### 2.1 Patients

From March 2018 to December 2019, a retrospective study of 784 consecutive patients who underwent conventional US and CEUS lung imaging was conducted at a tertiary pulmonary hospital. All patients were enrolled for the following reasons (1): a subpleural pulmonary lesion was detected by CT or magnetic resonance imaging (MRI); (2) The lesion was clearly visible on US imaging; (3) conventional US and CEUS were performed within 14 days prior to biopsy or surgery. The exclusion criteria were as follows: (1) a history of ipsilateral lung surgery, radiotherapy, chemotherapy, or neoadjuvant chemotherapy; (2) indefinite histopathological results; (3) final histopathological findings not LUAD or LUSC; (4) Incomplete clinical data or ultrasound images.

**Figure 1:**
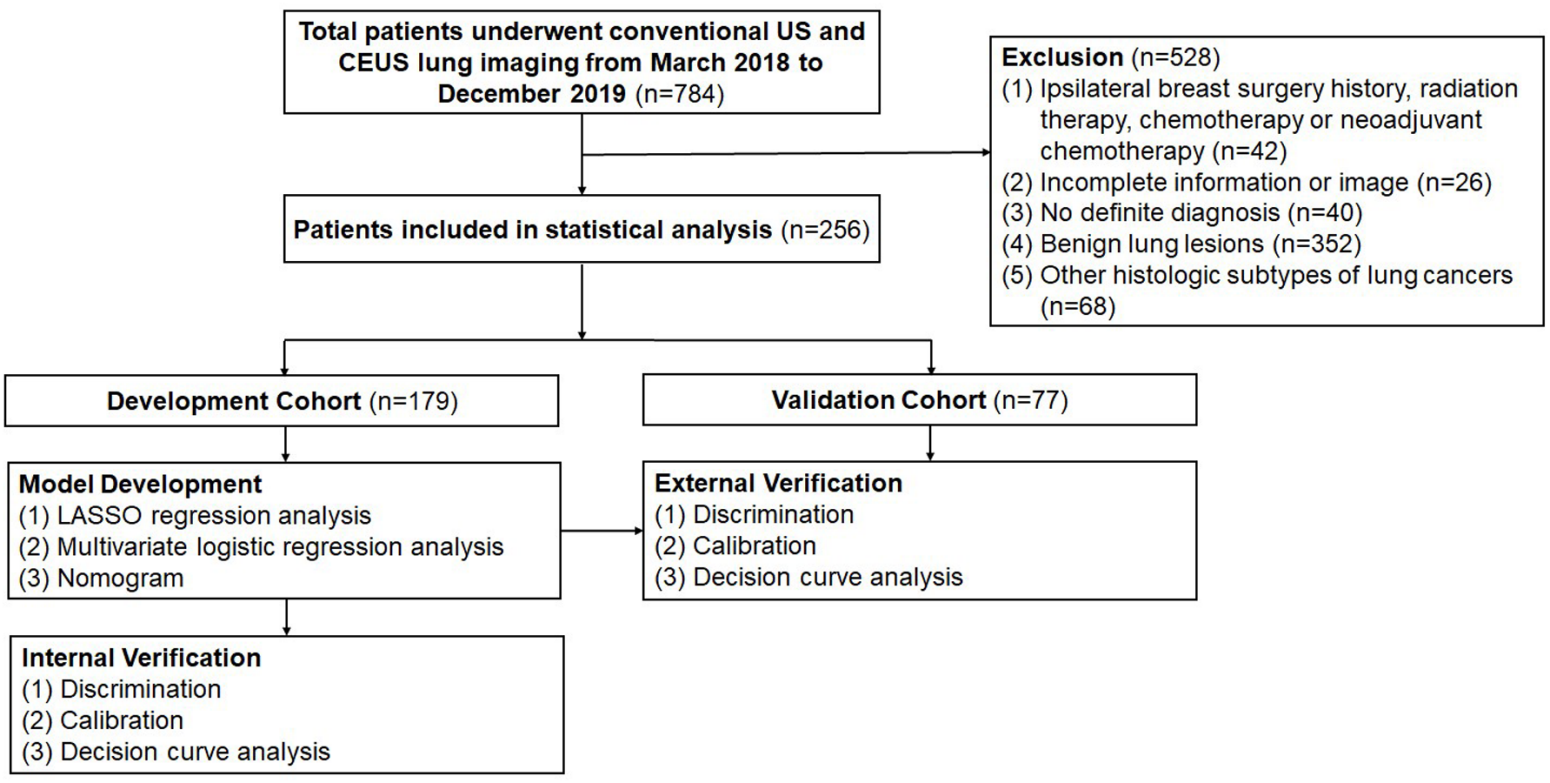
Flow diagram of study design. US, ultrasound; CEUS, contrast-enhanced ultrasound; LASSO, least absolute shrinkage and selection operator; AUC, area under the receiver operating characteristic curve.

Finally, 256 patients diagnosed with either LUAD or LUSC were included in the study. A randomized 7:3 classification scheme was employed to assign 179 patients to the development cohort (DC), which was used to establish a predictive model. Meanwhile, 77 patients were included in the validation cohort (VC), which was used to assess the model’s performance (**Figure 1**).

### 2.2 Serum sample collection

The results of serum carcinoembryonic antigen (CEA), CYFRA21-1, and squamous cell carcinoma antigen (SCC) tests conducted within 14 days prior to biopsy or surgery were retrospectively collected from the hospital’s electronic medical records for each patient. These results were then analyzed according to the manufacturer’s recommendations. Levels above the threshold were considered positive. Normal threshold for each marker was the following: CEA ≤ 10 ng/mL, CYFRA21-1 ≤ 3.3 ng/mL, SCC ≤ 3 ng/mL.

### 2.3 Image acquisition and analysis

Both conventional US and CEUS examinations were performed using the LOGIQ E9 US scanner (General Electric Healthcare, Chicago, IL, USA) with a 1–6 MHz convex probe. Two radiologists (M.J.S. and Y.Z.; both with 5 years of experience in lung US) performed the US examinations independently, with the understanding that they were blinded to all clinical information except for the location of the lesion. First, conventional US imaging was performed. The skin was coated with an adequate amount of coupling agent, and the probe was positioned parallel to the intercostal space. Uniform pressure was applied to provide the most comprehensive view of the lesion. The images were then stored in a dynamic format for further assessment. CEUS imaging was subsequently performed in a low mechanical index (0.1) contrast-enhanced mode. The gain was set to display only the surface of the air-filled lung (20 dB). Subsequently, 2.4 mL of US contrast agent (SonoVue; Bracco SpA, Milan, Italy) was injected into the cubital vein via a 20-gauge needle within 2 seconds, followed by a rapid flush with 5 mL of normal saline. The dynamic clip was recorded for 3 minutes, which was deemed sufficient to capture the dynamic enhanced features of the lesions (17).

On US images, the lesion was evaluated for size (largest diameter), shape (roundish or irregular), angle between lesion border and thoracic wall (obtuse or acute), margin with normal lung (regular or irregular), echogenicity (isoechoic or hypoechoic), homogeneity (homogeneous or heterogeneous), necrosis (presence or absence), air bronchogram (presence or absence), thoracic wall invasion (presence or absence, with presence including disruption of the pleura and fixation of tumor during respiration), and Adler grade of blood flow (0, 1, 2 or 3 grade) (18).

Qualitative CEUS parameters were obtained from CEUS images by analyzing dynamic clips frame by frame (1): perfusion pattern (hilum-to-pleura, periphery-to-center or part-to-whole); (2) degree of enhancement (hyper-enhancement, iso-enhancement, or hypo-enhancement, with hyper-enhancement defined as close to the enhanced degree of air-filled lung tissues, and hypo-enhancement defined as close to the enhanced degree of thoracic wall muscle); (3) homogeneity of enhancement (homogeneous or heterogeneous); (4) vascular sign (presence or absence, with vascular sign defined as the earliest enhanced blood vessels in the lesion); (5) perfusion defect (presence or absence).

In the classification of lesion characteristics by US and CEUS, two radiologists assessed independently (Y.Z., M.J.S.; both with 5 years of experience in CEUS and lung US). In case of a disagreement, we have implemented a process of discussion to reach a consensus. If consensus cannot be achieved through discussion, a third independent radiologist (Y.W., 18 years of CEUS and lung US experience) is involved in the evaluation to make the final determination.

### 2.4 Reference standard

The final diagnosis was based on biopsy with US guidance or surgery. The samples were fixed in 10% neutral formalin, and subsequently stained with hematoxylin-eosin. Finally, one of 3 board-certified pathologists, each with more than 5 years of experience in lung pathology, analyzed the samples with immunohistochemistry to determine the nature of the lesions. They were all blinded to the US and CEUS results.

### 2.5 Statistical analyses

A random division of all patients into DC (70%) and VC (30%) was conducted. The DC was used to construct a nomogram and perform internal validation, while the VC was utilized for external validation. Categorical variables were presented as absolute numbers and percentages, while continuous data were expressed as mean ± standard deviation or median and interquartile range (IQR). Continuous variables were compared using the Student’s t-test or the Mann-Whitney U test, while the chi-square (*χ*2) test or the Fisher’s exact test was performed to compare categorical variables.

Least absolute shrinkage and selection operator (LASSO) regression analysis was initially performed to identify the candidate variables exhibiting significant differences between LUAD and LUSC, utilizing a 10-fold cross-validation. The candidate variables were subsequently analyzed using a multivariate logistic regression analysis (backward stepwise method). Based on the independent prognostic factors, a nomogram was finally constructed. The model was used to calculate the probability of each case, and the receiver operating characteristic (ROC) curve was plotted to determine the cutoff value at the maximum Youden index.

The area under the receiver operating characteristic curve (AUC) was used to evaluate the discrimination. The calibration curve based on 1000 bootstrap re-samples was used to assess the calibration of the model, accompanied by the Hosmer-Lemeshow test. The clinical utility of the model was evaluated by decision curve analysis (DCA).

SPSS V.20.0 (SPSS Inc., Chicago, IL, USA) and R software V.4.2.1 (Institute for Statistics and Mathematics, Vienna, VIC, Austria) were used for the statistical analyses. The level of statistical significance was set at *p* < 0.05.

## 3 Results

### 3.1 Clinical and ultrasound characteristics

The 256 patients included 179 men and 77 women with a median age of 66 years (range, 62-72 years). Of the 256 lung lesions, 147 were LUAD, and 109 were LUSC. The 256 patients were divided into two groups: 179 in the DC and 77 in the VC. There were no significant differences between the DC and VC in terms of baseline clinical data, laboratory parameters, and US characteristics, except for homogeneity in conventional US (*p* = 0.031) (**Table 1**).

**Table 1:** Baseline characteristics of LUAD and LUSC patients in development cohort and validation cohort.

Characteristics	DC (n=179)				VC (n=77)			*p* Value^a^
	LUAD (n=102)	LUSC (n=77)		*p* Value	LUAD (n=45)	LUSC (n=32)	*p* Value	
Age (y)	65 (58,72)	70 (65,73)		0.009	65 (59,69)	69 (64,75)	0.031	0.700
Gender (n, %)				<0.001			0.004	0.844
Male	52 (51.0)	72 (93.5)			26 (57.8)	29 (90.6)		
Female	50 (49.0)	5 (6.5)			19 (42.2)	3 (9.4)		
Location (n, %)				0.782			0.029	0.604
Left	47 (46.1)	33 (42.9)			13 (28.9)	18 (56.2)		
Right	55 (53.9)	44 (57.1)			32 (71.1)	14 (43.8)		
Smoking history (n, %)				0.005			0.073	0.130
Yes	32 (31.4)	41 (53.2)			19 (42.2)	21 (65.6)		
No	70 (68.6)	36 (46.8)			26 (57.8)	11 (34.4)		
Serum tumor marker								
CEA (n, %)				<0.001			0.017	0.255
≤10 ng/mL	48 (47.1)	65 (84.4)			27 (60.0)	28 (87.5)		
>10 ng/mL	54 (52.9)	12 (15.6)			18 (40.0)	4 (12.5)		
CYFRA 21-1 (n, %)				0.024			0.646	0.702
≤3.3 ng/mL	49 (48.0)	27 (35.1)			19 (42.2)	11 (34.4)		
>3.3 ng/mL	53 (52.0)	50 (64.9)			26 (57.8)	21 (65.6)		
SCC (n, %)				0.177			0.567	0.369
≤3 ng/mL	102 (100.0)	74 (96.1)			44 (97.8)	30 (93.8)		
>3 ng/mL	0 (0.0)		3 (3.9)		1 (2.2)	2 (6.2)		
Conventional US								
Maximum diameter (cm)	5.2 (3.7,6.4)		6.4 (4.8,8.1)	<0.001	5.1 (3.1,6.4)	5.5 (4.5,7.1)	0.040	0.295
Shape (n, %)				0.349			0.685	0.352
Roundish	42 (41.2)		38 (49.4)		22 (48.9)	18 (56.2)		
Irregular	60 (58.8)		39 (50.6)		23 (51.1)	14 (43.8)		
Angle between lesion border and thoracic wall (n, %)				0.316			0.395	1.000
Obtuse	9 (8.8)		3 (3.9)		4 (8.9)	1 (3.1)		
Acute	93 (91.2)		74 (96.1)		41 (91.1)	31 (96.9)		
Margin with normal lung (n, %)				0.513			0.988	0.338
Regular	67 (65.7)		55 (71.4)		28 (62.2)	19 (59.4)		
Irregular	35 (34.3)		22 (28.6)		17 (37.8)	13 (40.6)		
Echogenicity (n, %)				0.094			1.000	0.608
Hypoechoic	73 (71.6)		45 (58.4)		32 (71.1)	22 (68.8)		
Isoechoic	29 (28.4)		32 (41.6)		13 (28.9)	10 (31.2)		
Homogeneity (n, %)				0.024			0.063	0.031
Homogeneous	57 (55.9)		29 (37.7)		33 (73.3)	16 (50.0)		
Heterogeneous	45 (44.1)		48 (62.3)		12 (26.7)	16 (50.0)		
Necrosis (n, %)				0.013			0.018	0.274
Yes	9 (8.8)		18 (23.4)		1 (2.2)	6 (18.8)		
No	93 (91.2)		59 (76.6)		44 (97.8)	26 (81.2)		
Air bronchogram (n, %)				0.884			0.269	0.524
Yes	42 (41.2)		30 (39.0)		13 (28.9)	14 (43.8)		
No	60 (58.8)		47 (61.0)		32 (71.1)	18 (56.2)		
Thoracic wall invasion (n, %)				0.602			0.163	0.635
Yes	27 (26.5)		24 (31.2)		8 (17.8)	11 (34.4)		
No	75 (73.5)		53 (68.8)		37 (82.2)	21 (65.6)		
Adler grade of blood flow (n, %)				0.227			0.788	0.764
0 grade	30 (29.4)		15 (19.5)		16 (35.6)	8 (25.0)		
1 grade	37 (36.3)		31 (40.3)		16 (35.6)	12 (37.5)		
2 grade	28 (27.5)		20 (26.0)		10 (22.2)	9 (28.1)		
3 grade	7 (6.9)		11 (14.3)		3 (6.7)	3 (9.4)		
CEUS								
Perfusion pattern (n, %)				0.102			1.000	0.848
Hilum-to-pleura	44 (43.1)		32 (41.6)		20 (44.4)	15 (46.9)		
Pleura-to-hilum	9 (8.8)		15 (19.5)		7 (15.6)	4 (12.5)		
Part-to-whole	49 (48.0)		30 (39.0)		18 (40.0)	13 (40.6)		
Degree of enhancement (n, %)				0.697			0.470	0.760
Hyper-enhancement	80 (78.4)		59 (76.6)		33 (73.3)	27 (84.4)		
Iso-enhancement	22 (21.6)		17 (22.1)		11 (24.4)	5 (15.6)		
Hypo-enhancement	0 (0.0)		1 (1.3)		1 (2.2)	0 (0.0)		
Homogeneity (n, %)				0.007			0.018	0.961
Homogeneous	32 (31.4)		10 (13.0)		16 (35.6)	3 (9.4)		
Heterogeneous	70 (68.6)		67 (87.0)		29 (64.4)	29 (90.6)		
Local later enhancement (n, %)				0.265			0.154	0.793
Yes	6 (5.9)		9 (11.7)		1 (2.2)	4 (12.5)		
No	96 (94.1)		68 (88.3)		44 (97.8)	28 (87.5)		
Vascular sign (n, %)				0.080			0,254	0.998
Yes	49 (48.0)		48 (62.3)		21 (46.7)	20 (62.5)		
No	53 (52.0)		29 (37.7)		24 (53.3)	12 (37.5)		
Perfusion defect (n, %)				<0.001			0.002	0.643
Yes	34 (33.3)		57 (74.0)		14 (31.1)	22 (68.8)		
No	68 (66.7)		20 (26.0)		31 (68.9)	10 (31.2)		

LUSC, lung squamous cell carcinoma; LUAD, lung adenocarcinoma; DC, development cohort; VC, validation cohort; CEA, carcinoembryonic antigen; SCC, squamous cell carcinoma antigen; US, ultrasound; CEUS, contrast-enhanced ultrasound.

a Data are compared between the DC and VC.

### 3.2 LASSO and logistic regression of development cohort

**Figure 2:**
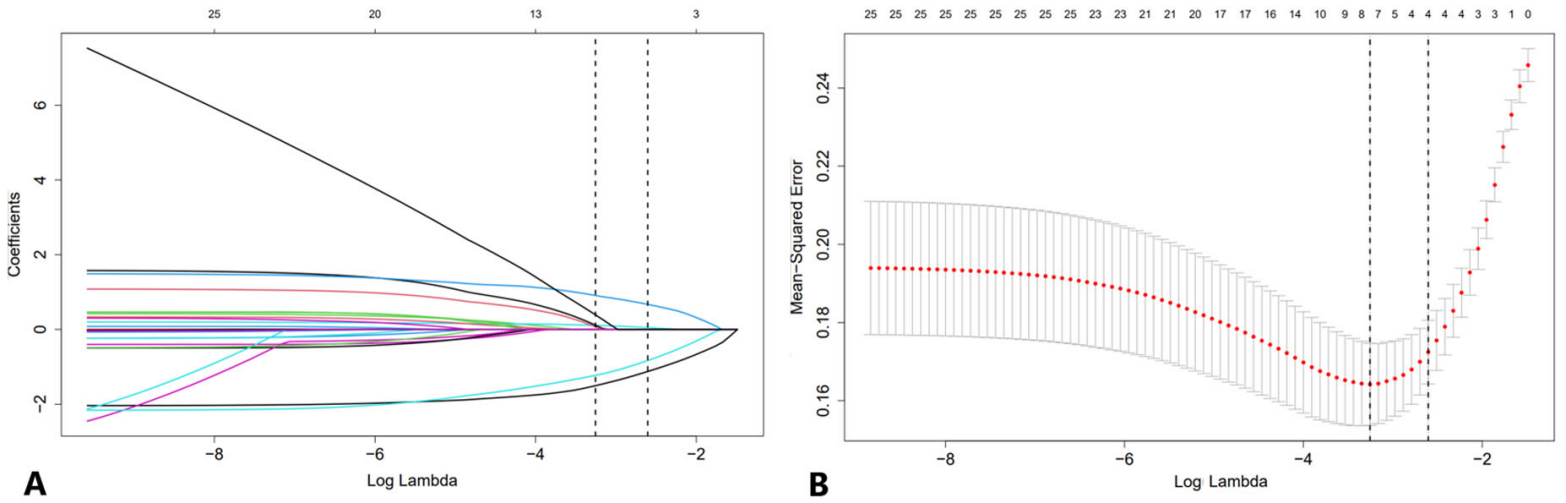
LASSO regression analysis with 10-fold cross-validation for candidate variable identification. **(A)** A plot of the coefficient profile against the log (lambda) sequence. **(B)** Tuning parameter (lambda) selection of the deviation in the LASSO regression based on the minimum criteria (left dotted line) and the 1 standard error criteria (right dotted line). In this study, the predictor was selected according to the 1 standard error criteria, where 4 non-zero coefficients were selected. LASSO, least absolute shrinkage and selection operator.

**Figure 3:**
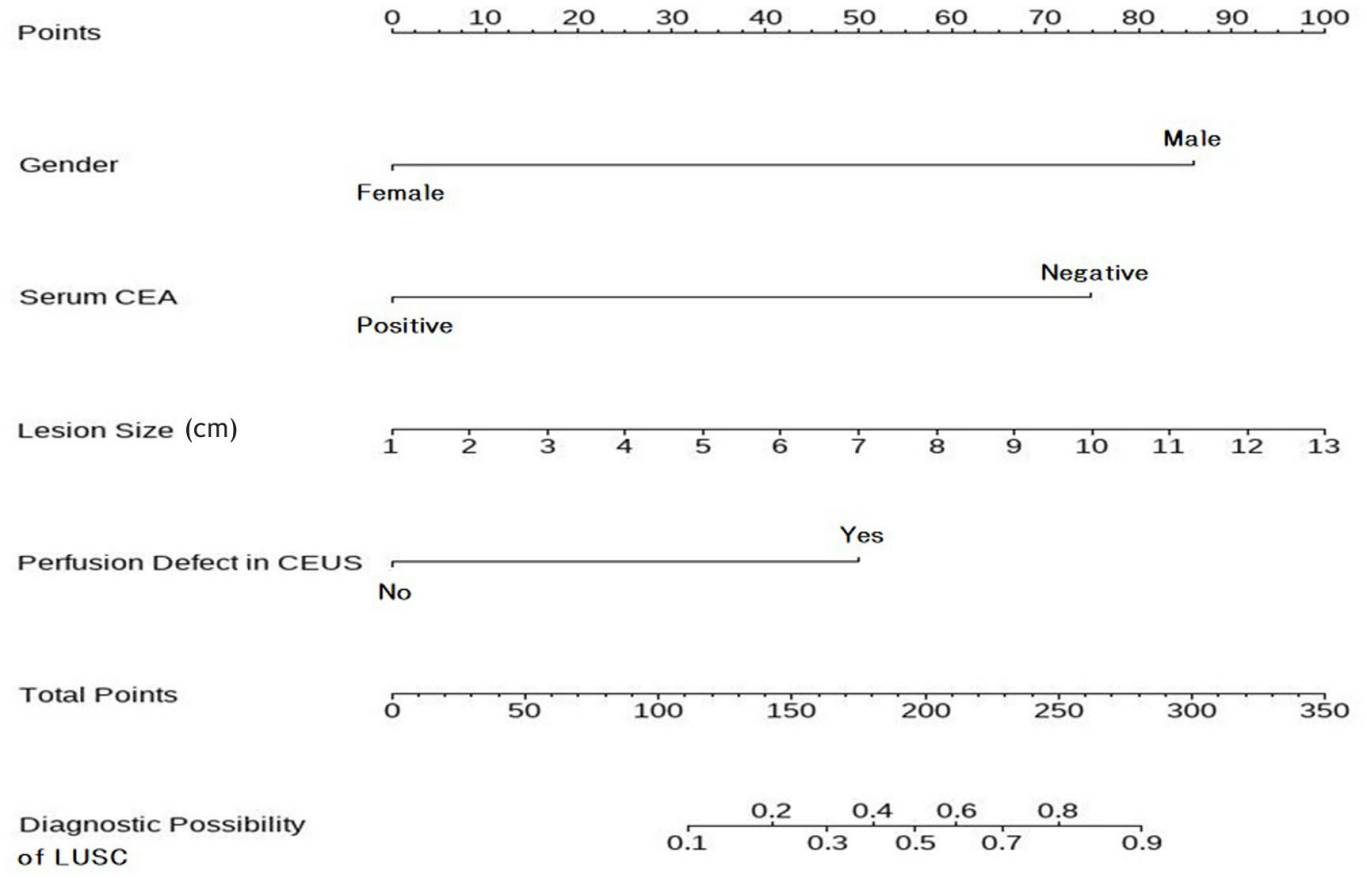
Nomogram to predict the histologic subtypes of subpleural NSCLC. To utilize the nomogram, begin by determining the value of each risk factor associated with an individual lesion, which can be found on the corresponding axis. Draw a line up to the horizontal Points axis at the top of the nomogram and record the corresponding points. The sum of these points can then be located on the horizontal Total Points axis at the bottom of the nomogram. Finally, a line should be drawn further down to the Predictive Probability axis, which will allow the probability of LUSC to be determined. NSCLC, non-small cell lung cancer; LUSC, lung squamous cell carcinoma.

**Figure 4:**
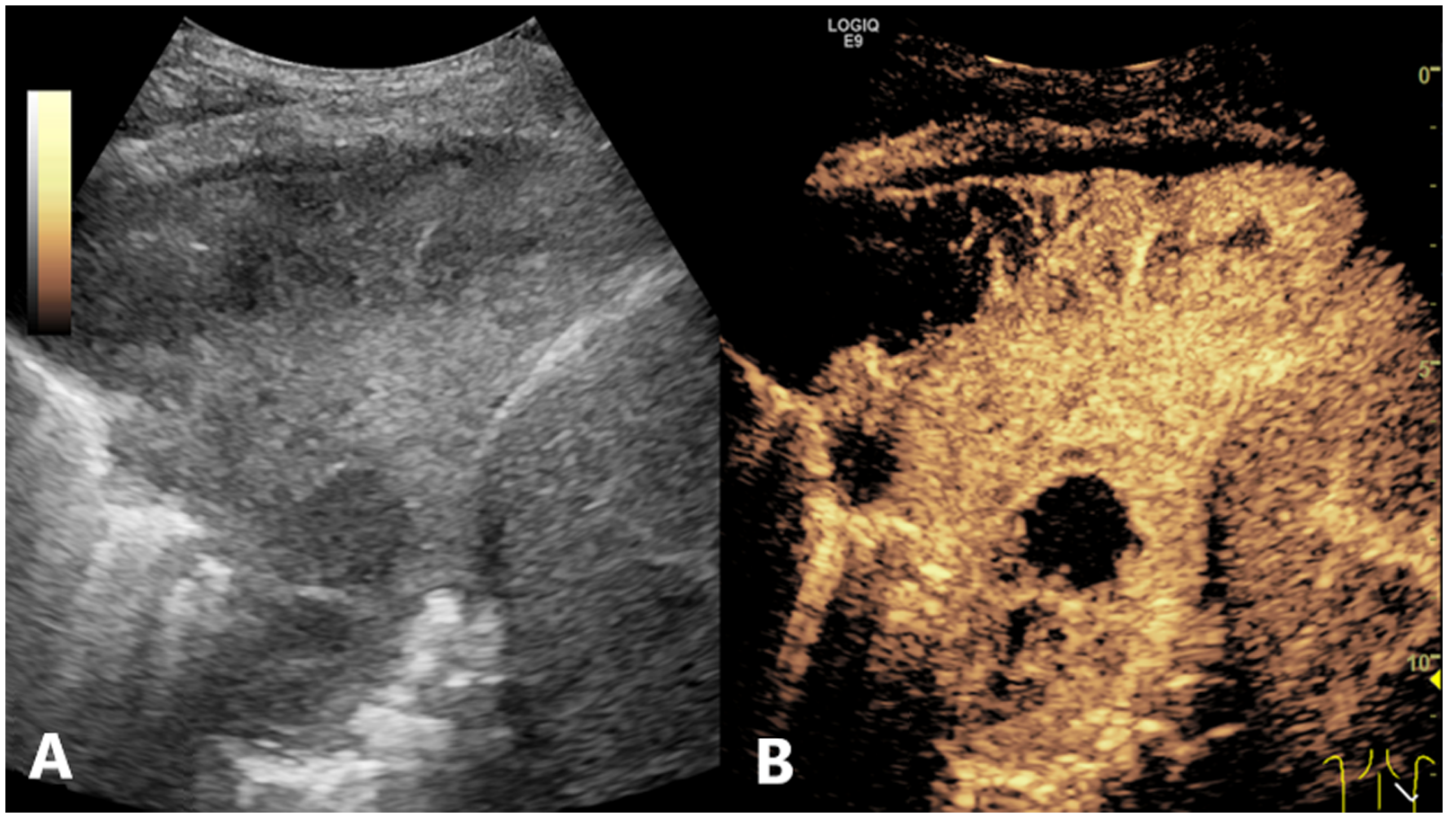
Conventional US and CEUS images of a subpleural pulmonary lesion (LUSC) in the lower lobe of right lung of a 62-year-old male. Conventional US **(A)** showed an isoechoic lesion with a maximum diameter of 9.8 cm. The lesion exhibited an irregular shape and a heterogeneous composition. CEUS **(B)** demonstrated a heterogeneous pattern of hyper-enhancement in the lesions, accompanied by the presence of perfusion defects. The serum CEA result was negative. The probability of LUSC, as calculated by the prediction model, was approximately 0.9, which was greater than the cutoff value (0.375). This indicated that the lesion was predicted to be LUSC, which was consistent with the definitive diagnosis. US, ultrasound; CEUS, contrast-enhanced ultrasound; LUSC, lung squamous cell carcinoma; CEA, carcinoembryonic antigen.

**Figure 5:**
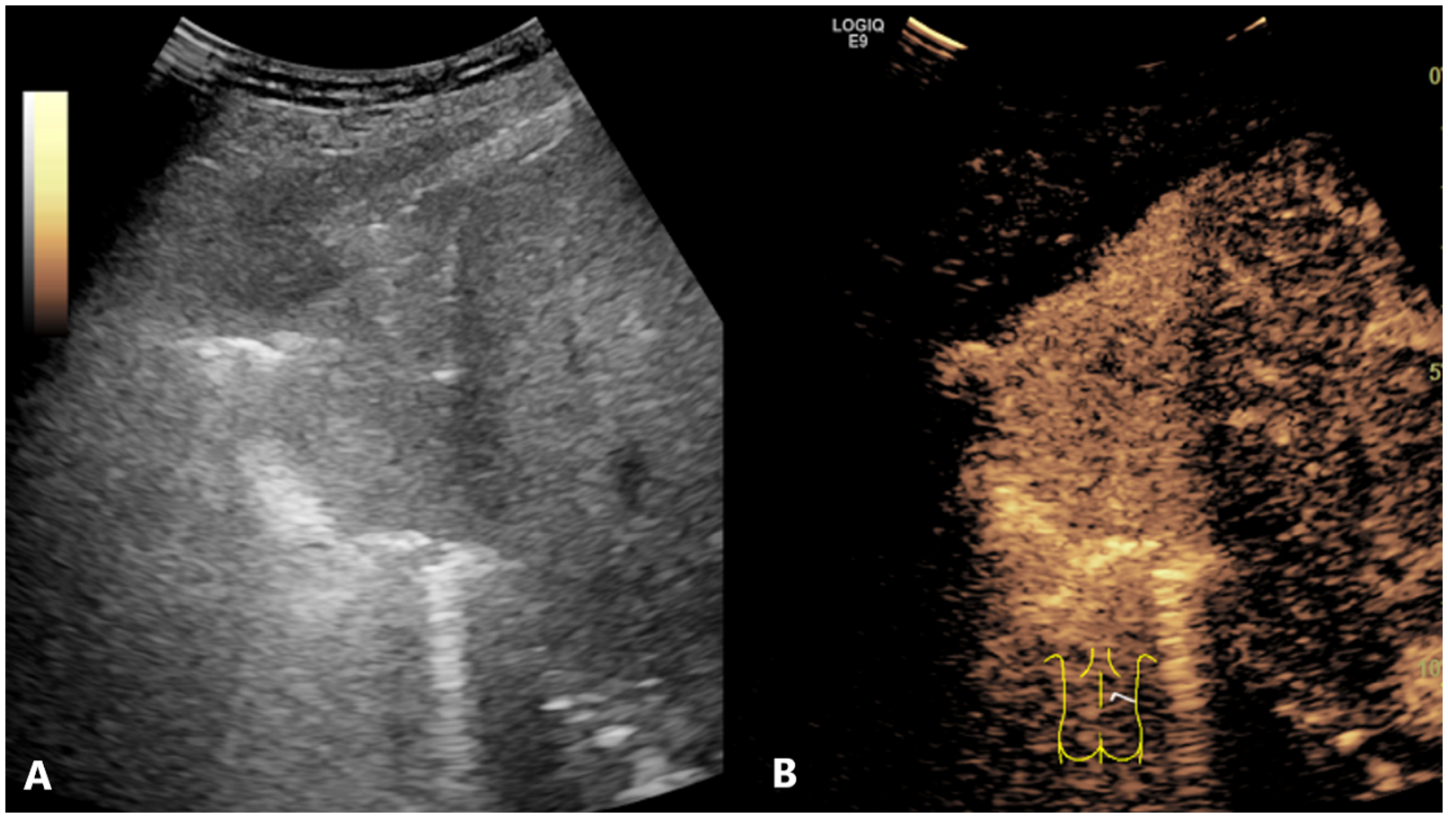
Conventional US and CEUS images of a subpleural pulmonary lesion (LUAD) in the lower lobe of right lung of a 64-year-old female. Conventional US **(A)** revealed an isoechoic lesion with a maximum diameter of 6.3 cm. The lesion exhibited an irregular shape and a homogeneous composition. CEUS **(B)** displayed a homogeneous pattern of hyper-enhancement in the lesions, accompanied by the absence of perfusion defects. The serum CEA result was negative. The probability of LUSC, as calculated by the prediction model, was approximately 0.1, which was less than the cutoff value (0.375). This indicated that the lesion was predicted to be LUAD, which was consistent with the definitive diagnosis. US, ultrasound; CEUS, contrast-enhanced ultrasound; LUAD, lung adenocarcinoma; LUSC, lung squamous cell carcinoma; CEA, carcinoembryonic antigen.

The LASSO path diagram showed that as the coefficients decreased, the number of predictors decreased accordingly (**Figure 2A**). Four predictor variables were selected based on lambda.1se in **Figure 2B**: gender, serum CEA, lesion size, and perfusion defect in CEUS. A multivariate logistic regression was then performed on the above variables to establish the predictive model, which is presented in **Table 2**. As all four predictors exhibited statistically significant differences, they were introduced into the final predictive model to develop a quantitative prediction nomogram for discriminating between LUAD and LUSC in NSCLC patients (**Figure 3**). The applications of the model in LUAD and LUSC are shown in **Figures 4**, **5**.

**Table 2:** Multivariate analysis of candidate variables derived from the development cohort.

Intercept and variables	*β*	*S.E.*	*OR*	95% *CI*	*z* Value	*p* Value
Intercept	1.095	0.830	2.989	0.598-15.890	1.320	0.187
Gender	-2.222	0.548	0.108	0.033-0.295	-4.052	<0.001
Serum CEA	-1.935	0.448	0.144	0.057-0.336	-4.320	<0.001
Lesion size	0.215	0.090	1.240	1.046-1.494	2.385	0.017
Perfusion defect in CEUS	1.292	0.417	3.639	1.624-8.387	3.102	0.002

*β*, regression coefficient; S.E., standard error; OR, odds ratio; CI, confidence interval; CEA, carcinoembryonic antigen; CEUS, contrast-enhanced ultrasound.

### 3.3 Predictive model validation

**Figure 6:**
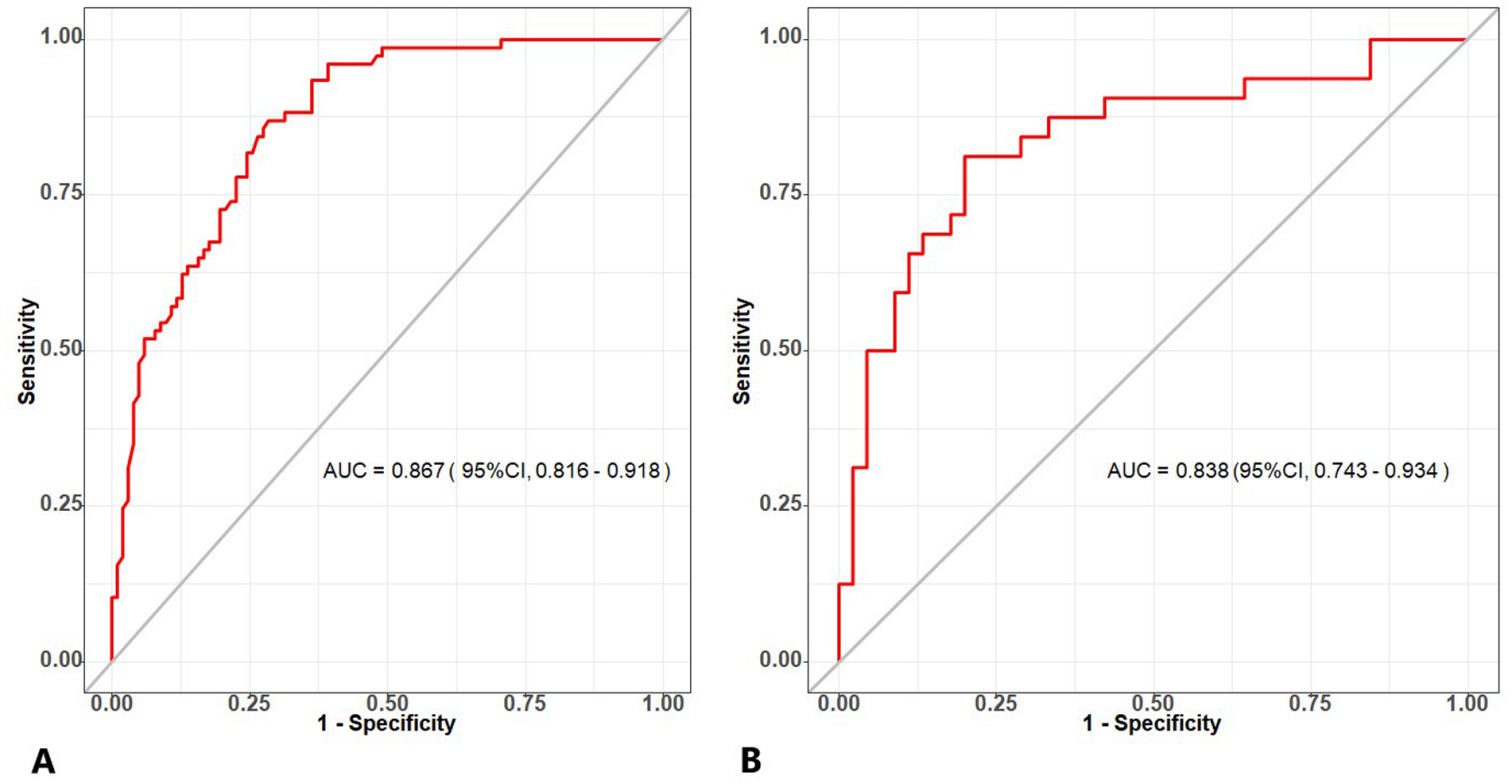
**(A)** The ROC curve of the combined model generated from the development cohort. **(B)** The ROC curve of the combined model generated from the validation cohort. ROC, receiver operating characteristic.

AUC values were calculated to assess the discrimination of the predictive model for differentiating the histologic subtypes of subpleural NSCLC in the DC and VC. As illustrated in **Figure 6**, the predictive model yielded an AUC value of 0.867 [95% confidence interval (CI)=0.816-0.918] in the DC, and an AUC value of 0.838 (95% CI=0.743-0.934) in the VC. The optimal cutoff for the nomogram in the DC was 0.375, with a sensitivity of 0.716 and a specificity of 0.870. In the VC, the sensitivity and specificity were 0.800 and 0.838, respectively. These data indicate that the nomogram has good discriminatory ability and predictive value.

**Figure 7:**
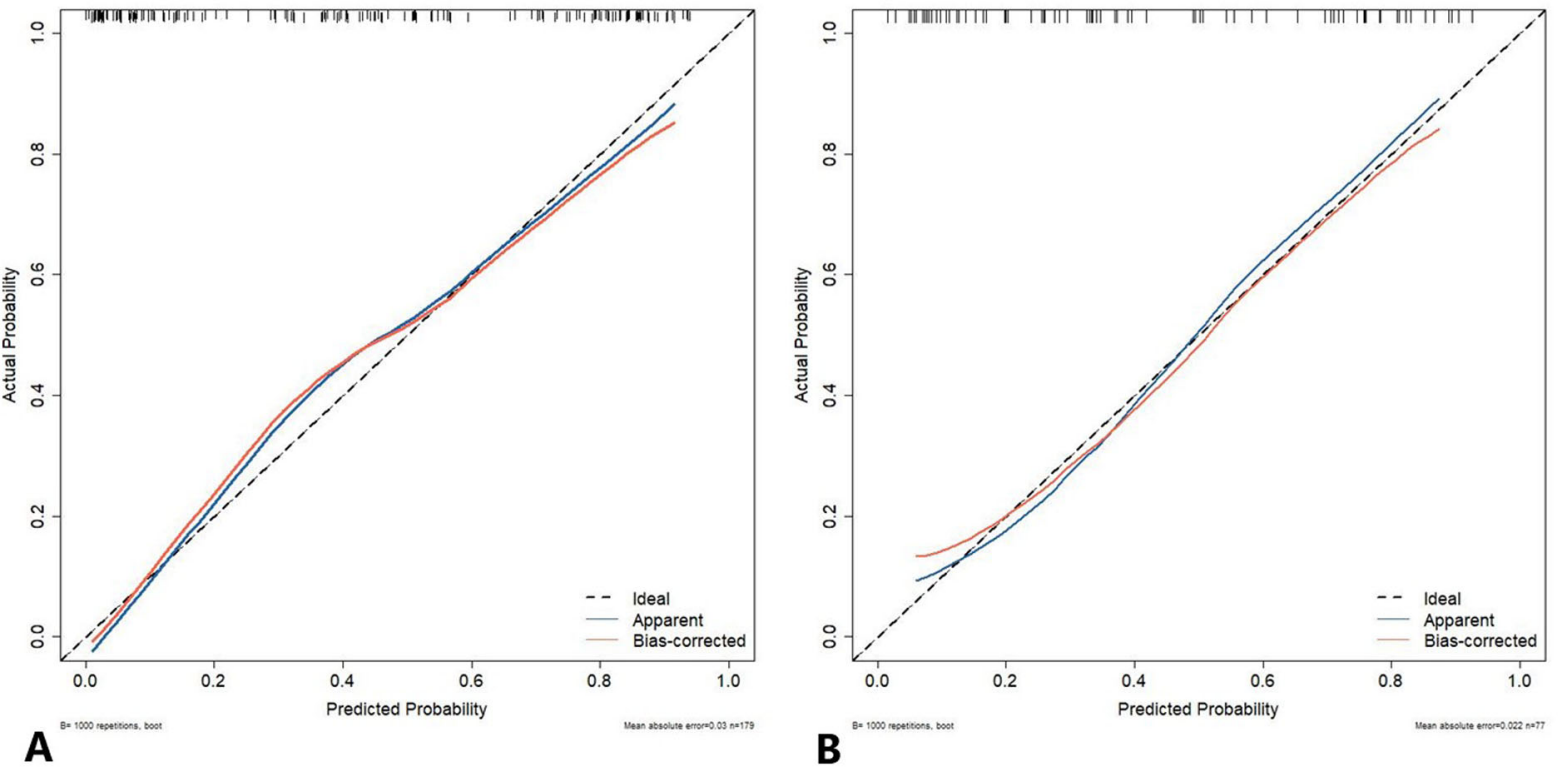
The calibration curve of the nomogram prediction model. The y-axis represents the actual diagnosed cases of LUSC, while the x-axis represents the predicted risk of LUSC. The diagonal dotted line represents an optimal prediction resulting from an ideal model. The solid line represents the observed performance of the development cohort **(A)** and validation cohort **(B)**, with the results indicating that a closer fit to the diagonal dotted line represents a more accurate prediction. LUSC, lung squamous cell carcinoma.

The calibration curve of the nomogram prediction model demonstrated a good consistency between the prediction by the nomogram and the actual observation in both DC and VC (**Figure 7**). As shown by the Hosmer-Lemeshow test, the predicted and actual probability were highly consistent in both DC (*p* = 0.525) and VC (*p* = 0.460).

**Figure 8:**
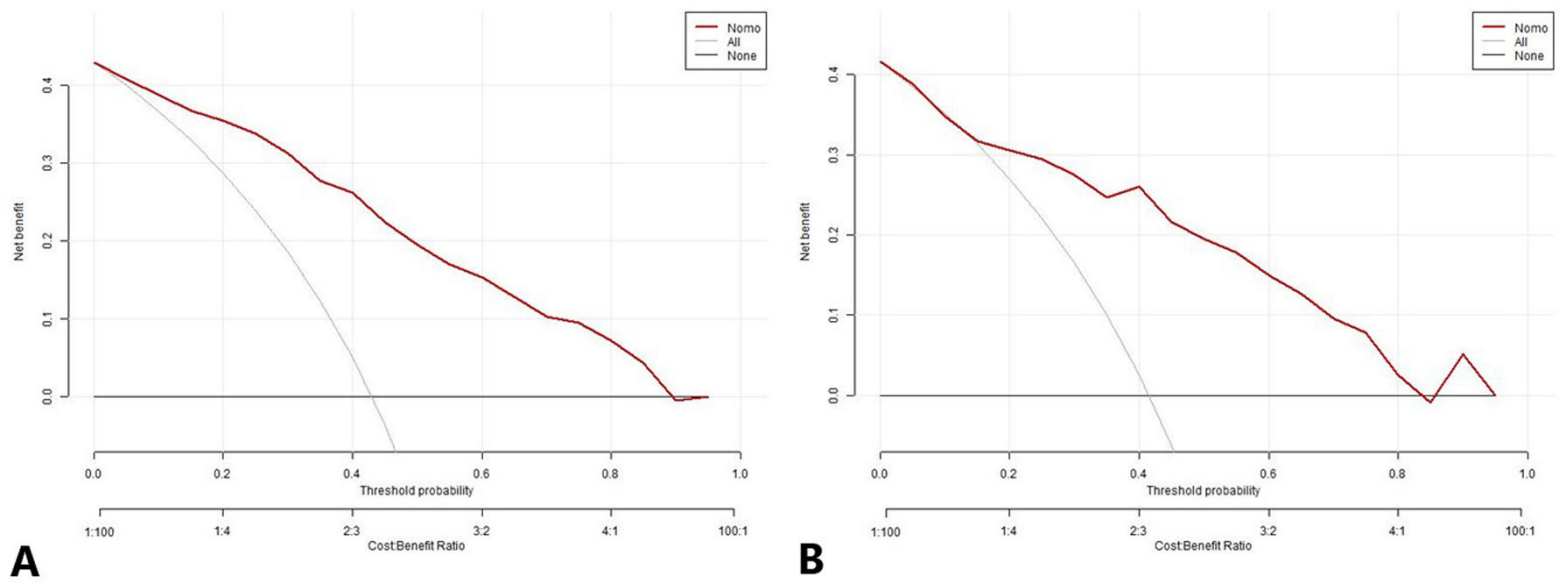
The decision curve analysis for the nomogram prediction model in the development cohort **(A)** and validation cohort **(B)**. The x-axis and y-axis represent the threshold probability and net benefit, respectively. The black line represents the hypothesis that all patients have LUAD, the light gray line represents the hypothesis that all patients have LUSC, and the red line represents the risk nomogram. The area between the black line and light gray line in the model curve represents the clinical utility of the model. The greater the distance between the model curve and the black and light gray lines, the more effective the clinical utility of the nomogram. LUAD, lung adenocarcinoma; LUSC, lung squamous cell carcinoma.

The DCA also indicated that the prediction model was a reliable clinical management tool for predicting the histologic subtypes in NSCLC when the risk threshold was 2-96% in the DC and 14-87% in the VC. (**Figure 8**).

## 4 Discussion

It should be acknowledged that chest CT has emerged as an important modality for the screening of lung cancer (19). Nevertheless, the requisite instrumentation for such diagnostic evaluations is not universally accessible, particularly within the confines of smaller, peripherals hospitals. Moreover, the procedural costs associated with CT are consistently higher in comparison to those of ultrasound examinations. The cumbersome nature of CT machinery further complicates its mobility. Additionally, the inherent risk of radiation exposure poses a significant concern. On a different note, there is a cadre of patients who exhibit allergic reactions to CT contrast agents. In such scenarios, where lung lesions are in close proximity to the chest wall, ultrasound may present a viable alternative diagnostic method. Ultrasound can rapidly and accurately identify subpleural lesions when coupled with CT. In regions where CT is not available, however, anterior and lateral chest X-rays may serve as a suboptimal alternative, with the reliability of this approach depending on the experience of the operator. For cases where the diagnosis remains indeterminate following enhanced CT, CEUS can provide valuable supplementary or combinatorial diagnostic insights (20). Furthermore, CEUS-guided biopsy has demonstrated high degrees of sensitivity and specificity in the pathological diagnosis of patients with peripheral lung cancers, thereby offering a reliable diagnostic alternative (21). The current literature indicates that ultrasound and contrast-enhanced ultrasound have a high diagnostic accuracy in differentiating benign and malignant lesions of peripheral lung cancer (22, 23). However, there is a paucity of studies investigating their potential for histologic classification of peripheral lung cancer (24).

Moreover, several literatures have demonstrated that the integration of clinical data with US imaging can significantly enhance the efficacy of diagnostic algorithms (25, 26). In this study, we successfully developed and validated a combined clinical-US model, which has good performance in noninvasively stratifying the histologic subtypes of NSCLC patients.

### 4.1 Association of gender with the histologic subtypes in NSCLC

Both DC and VC in our study indicated that, in comparison to LUAD, there were considerably more male patients with LUSC. This finding is consistent with previously published literature reports (27). Previous studies have demonstrated that LUSC is associated with smoking, and there is a discernible dose-response relationship between smoking and the occurrence of squamous cell carcinoma (28). Additionally, the intensity of smoking is higher in men, which may contribute to the observed prevalence of LUSC in this demographic. However, LUAD is less dependent on smoking habits, the effects of cooking fume exposure (29, 30) and hormone exposure (31–33) have been proposed as possible factors for the apparent increase in the proportion of women in LUAD.

### 4.2 Association of serum CEA with the histologic subtypes in NSCLC

Among the three serum tumor markers assayed in this study, only serum CEA was included in the final model as an independent predictor. The serum CEA level was observed to be approximately half positive in the LUAD group in both DC and VC, with a much lower prevalence of positivity in the LUSC group. CEA was the first serum tumor marker to be associated with lung cancer (34). The elevation of CEA in patients with tumors may be attributed to a shift in the expression of oncogenes (35). When cells undergo malignant transformation, the corresponding chromosome is epigenetically repressed, thereby enabling the previously silenced allele to reactivate in the tumor microenvironment and produce CEA. LUAD cells exhibit a high proliferative activity, which results in a significantly elevated CEA value in LUAD when compared to that observed in LUSC.

### 4.3 Association of lesion size with the histologic subtypes in NSCLC

The results of our study demonstrated that the mean maximum diameter of LUSC was significantly larger than that of LUAD. In most studies (15, 36, 37), a relatively smaller tumor size was found in LUAD patients than in LUSC patients, which is consistent with the natural history of the disease. LUSC achieves larger tumor volumes in the lung primary lesion before giving nodal and distant metastases. Moreover, all patients included in this study had peripheral lesions in close proximity to the chest wall, which could be observed by ultrasound. Due to the differing origins of the lesions, LUSC often exhibited a large invasion area when detected by ultrasound in the subpleural region.

### 4.4 Association of perfusion defect in CEUS with the histologic subtypes in NSCLC

This study demonstrated that the presence of a perfusion defect in contrast-enhanced ultrasound (CEUS) is an independent predictor of lung squamous cell carcinoma (LUSC). The contrast agent utilized in this study, SonoVue, is a blood pool tracer. CEUS can depict the distribution of microvasculature within the lesion, offering more details about the tumor perfusion than conventional ultrasound (38, 39). Although tumor vessels around the cancer nest are abundant, they are mostly fine blood vessels, lacking thick nourishing blood vessels to provide sufficient nutrients. LUSC tends to be too large and tumor tissue growth is relatively dense, resulting in local tissue pressure increases to compress the main tumor blood vessels. Consequently, blood supply is relatively insufficient within the lesion, leading to local ischemia and necrosis, which displayed a region of perfusion defect in CEUS. Notably, LUSC is prone to coagulation necrosis (40), with minimal distinction between the necrotic region and the adjacent non-necrotic region, rendering differentiation challenging in conventional ultrasound examinations. Following CEUS, the active area was filled with contrast agent, which demonstrated significant enhancement. In contrast, the contrast agent could not enter the necrotic region due to a lack of blood supply, which displayed perfusion defect. In this study, necrotic regions were identified by conventional ultrasound in 13.3% of the lesions, while they were identified by CEUS in 49.6% of the lesions, which fully confirmed this view.

The combination of clinical data and US parameters was proved to improve the diagnostic efficiency of predicting the histologic subtypes of subpleural NSCLC. The AUC of the combined model in DC and VC were 0.864 and 0.849, respectively. To date, no studies have been conducted that combine clinical data and US parameters in the context of lung cancer. Consequently, the results of the present study cannot be compared with those of previous studies. Wang et al. (24) proposed that if the “dead wood” pattern, a feature of microflow imaging in CEUS, were to be regarded as the diagnostic criterion for LUSC, its diagnostic sensitivity, specificity, and accuracy would be 62.9%, 93.3%, and 82.1%, respectively. The definition of microflow patterns indicated that necrosis was commonly observed in the “dead wood” pattern, while it was rarely shown in the other patterns. Thus, it is possible to argue that necrosis represents a key differentiation between LUSC and LUAD. Chen et al. (41) also demonstrated analogous distinctions in microflow patterns between LUSC and LUAD. Notably, the MFI pattern could not cover all types of peripheral lung cancers (24). Consequently, in this study, the presence or absence of perfusion defects in CEUS was employed to describe all the lesions. Significant differences were observed between LUSC and LUAD with regard to perfusion defects.

The Hosmer-Lemeshow test results, with p-values of 0.525 in the DC and 0.460 in the VC, confirmed a high degree of consistency between the predicted probabilities. Also, the diagnostic evaluation was carried out for our model through AUC, calibration curves, and DCA provides a comprehensive assessment of its performance and utility. It confirms that the model has good discriminatory ability, is well-calibrated, and has practical value for predicting histologic subtypes in NSCLC. Specifically, the proposed model demonstrates the capacity to furnish integrated or supplementary diagnostic data for cases in which pathological findings are inaccessible or the diagnostic outcome remains ambiguous, thereby enhancing the precision of clinical assessment.

### 4.5 Limitations

First, this is a single-center retrospective study, which carries the inherent risk of selection bias. Therefore, the findings must be validated by multi-center prospective studies. Second, the present study focused exclusively on LUSC and LUAD in NSCLC. It is noteworthy that other histological subtypes of NSCLC, such as sarcomatoid carcinoma, can also exhibit significant necrosis, although this accounts for a relatively small percentage of NSCLC cases. Third, it is not uncommon for lung tumors to be partially obscured by gas or ribs, which can result in the omission of pertinent lesion information during US analysis. Finally, the present study employs a qualitative and semi-quantitative approach to ultrasound evaluation. However, more objective measurement methods, such as quantitative contrast-enhanced ultrasound analysis and computer-aided diagnosis, have yet to be adopted. Consequently, these aspects of research require further exploration in future studies.

## 5 Conclusion

In this study, a nomogram has been developed that can predict the histologic subtypes of subpleural NSCLC with optimal discrimination and calibration. The tool has the potential to facilitate clinicians in making treatment recommendations.

## Data Availability

The raw data supporting the conclusions of this article will be made available by the authors, without undue reservation.
